# Pyrimidine metabolic rate limiting enzymes in poorly-differentiated hepatocellular carcinoma are signature genes of cancer stemness and associated with poor prognosis

**DOI:** 10.18632/oncotarget.20774

**Published:** 2017-09-08

**Authors:** Hsi-Wen Yeh, Szu-Shuo Lee, Chieh-Yu Chang, Chun-Mei Hu, Yuh-Shan Jou

**Affiliations:** ^1^ Graduate Institute of Life Sciences, National Defense Medical Center, Taipei, Taiwan; ^2^ Institute of Biomedical Sciences, Academia Sinica, Taipei, Taiwan; ^3^ Program in Molecular Medicine, National Yang-Ming University and Academia Sinica, Taipei, Taiwan; ^4^ Taiwan International Graduate Program in Molecular Medicine, National Yang-Ming University and Academia Sinica, Taipei, Taiwan; ^5^ Genomic Research Center, Academia Sinica, Taipei, Taiwan

**Keywords:** hepatocellular carcinoma, poor differentiation, pyrimidine metabolism, rate-limiting enzymes, stemness

## Abstract

Cellular metabolism of cancer cell is generally recognized to provide energy for facilitating tumor growth, but little is known about the aberrant metabolism in tumor progression and its prognostic value. Here, we applied integrated genomic approach to uncover the aberrant expression of metabolic enzymes in poorly-differentiated human hepatocellular carcinoma (HCC) for revealing targets against HCC malignancy. A total of 135 upregulated (22 are rate-limiting enzymes (RLEs)) and 362 down-regulated (77 are RLEs) metabolic genes were identified and associated with poor patient survival in large-cohorts of HCC patients in TCGA-LIHC and two other independent transcriptomic studies. Ten out of 22 upregulated RLEs in poorly-differentiated HCC are critical enzymes in pyrimidine metabolism pathways in association with stemness features by gene enrichment analysis and upregulated in ALDH1^+^ stem-like HCC subpopulations. By focusing on three RLEs including TK1, TYMS and DTYMK of dTTP biosynthesis pathway, expression of 3 RLEs in well-differentiated HCC cells increased ALDH1^+^ and spheroid stemness population but reversed by knockdown in poorly-differentiated HCC cells. Up-regulated 3 RLEs in HCC were associated with poor patient survival in multiple cohorts. Together, we identified aberrant pyrimidine pathway in poorly-differentiated HCC promotes cancer stemness served as potential theranostic target for battling HCC tumor progression.

## INTRODUCTION

Hepatocellular carcinoma (HCC), the major malignancy of liver, is the sixth most common cancer in the world and the second leading cause of cancer deaths for the last decade with around 0.8 million each of new cases and deaths annually [1]. Most successful HCC therapeutic options are surgical resection, transplantation and ablation therapy for only about 30% eligible early-stage patients, but limited efficacy to most of patients due to late-stage HCC at the time of diagnosis. The prognosis is very poor for untreated HCC patients with an average survival between 6 and 20 months [2, 3] and 40~80% of treated patients developed recurrence and metastasis within 5 years of therapy [4, 5]. Sorafenib, a multiple kinase inhibitor, is the only FDA-approved systemic treatment to advanced-stage HCC patients with a statistically significant increase of overall survival benefit by about 3 months [6, 7].

Reprogramming of cancer cell metabolism to fuel uncontrollable privilege of cell proliferation in compared with surrounding normal cells is emerging as the new hallmark of cancer [8]. Altered energy metabolism in cancer cell was first observed since 1930 by Otto Warburg that cancer cells are favored to increase glucose uptake and utilize less efficient ATP producing aerobic glycolysis in compared to the high ATP producing glucose oxidative phosphorylation in mitochondria in the normal cells [9]. Depending on the intrinsic genetic background of cancer cells and extrinsic nutrient availability and cellular interactions in the tumor microenvironment, altered cellular metabolism could support anabolic growth of cancer cells in the nutrient-rich environment, catabolic supports of cancer cell survival under limited nutrient, and fortification of redox imbalance to facilitate oncogene activation, tumor suppressor loss, and other tumorigenic stresses [10, 11]. Recent cancer genomic data revealed that different somatic mutations of metabolic genes in different cancer types such as loss of function of mutated succinate dehydrogenase (SDH) or fumarate hydratase (FH) in certain renal cell carcinomas and mutated isocitrate dehydrogenase (IDH) 1 or 2 in glioma, acute myeloid leukemias, chondrosarcomas, and amplification of phosphoglycerate dehydrogenase (PHGDH) in estrogen receptor (ER)-negative breast cancer and melanoma further suggest that somatic alterations in metabolism could satisfy cancer-specific demands for fueling tumor growth [12–16]. Although it is generally recognized that altered cellular metabolism of cancer cells could facilitate cell proliferation and transformation, little is known about the metabolic changes that promote cancer cell aggressiveness [17–20].

Cancer cell aggressiveness is tightly associated with features of epithelial to mesenchymal transition (EMT), stemness, poor differentiation and high mobility of cancer cells leading to outcomes of drug resistance, recurrence and metastasis resulted in poor survival of cancer patients [21, 22]. To reveal how metabolic reprogramming contributes to aggressiveness and serves as theranostic target during tumor progression, we examined the altered expression of metabolic enzymes in association with histopathological feature of poor differentiation of HCC using the large number of HCC patients from TCGA cohorts. Interestingly, we identified a unique pyrimidine metabolic rate limiting enzymes (RLEs) gene signature that is altered in poorly- differentiated HCC and correlated to the stemness of embryonic signatures and poor patient survival. With validations of experiments and HCC patients in multiple cohorts, we provided lines of evidence that TK1, TYMS and DTYMK the catalytic RLEs in pyrimidine biosynthesis play critical roles in cancer stemness and serve as potential therapeutic targets in poorly-differentiated HCC.

## RESULTS

### A unique metabolic gene expression signature in poorly-differentiated hepatocellular carcinoma patients

**Figure 1:**
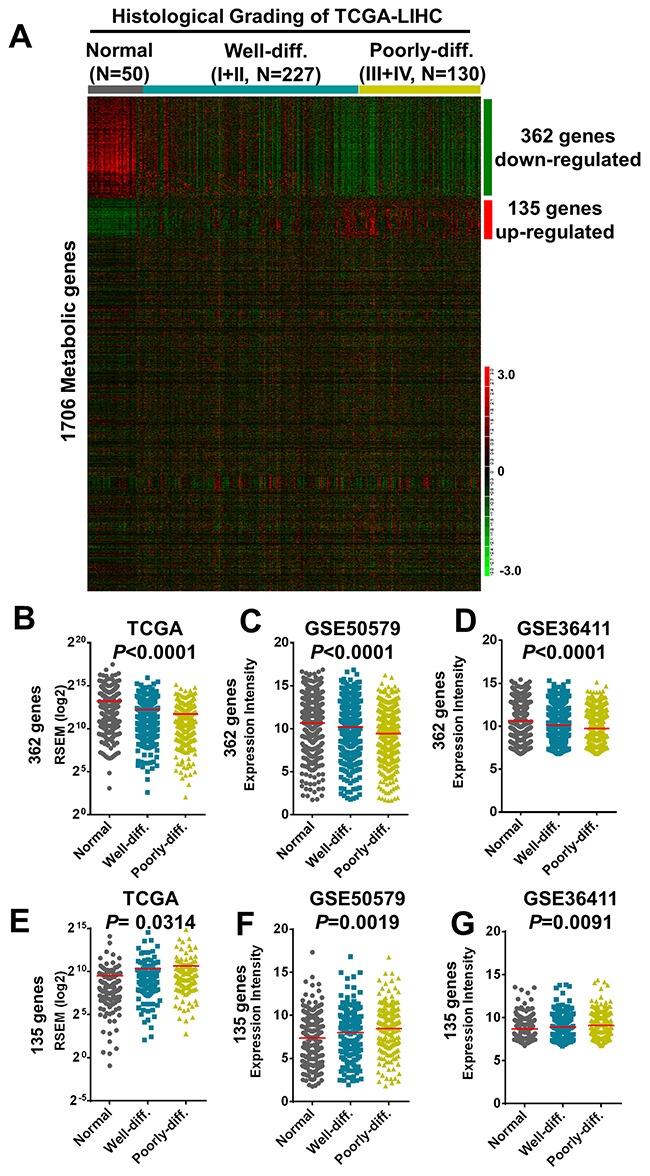
Transcriptomic correlations of 1706 metabolic genes with histological grading of human hepatocellular carcinoma in TCGA and microarray datasets **(A)** A total of 497 including 362 downregulated and 135 upregulated genes were revealed after clustered with histological differentiation of HCC tumors as shown in the heatmap. **(B-G)** The 362 downregulated genes clustered with differentiation status of HCC tissues was shown in Sum RSEM of TCGA-LIHC (B) and validated by microarray datasets of GSE50579 (C) and GSE36411 (D). The135 upregulated genes were shown and validated in the same datasets (E-G), respectively. Statistical analysis was performed in one-way ANOVA.

To determine the involvement of metabolic genes during tumor progression of HCC especially focusing on tumor differentiation, we downloaded RNA transcriptomic datasets performed by next generation sequencing (RNA-seq) in The Cancer Genome Atlas (TCGA) project (TCGA-LIHC) containing 50 normal liver tissues and 357 HCC samples including 227 well-differentiated HCC (Grade I and II of histological grading) and 130 poorly-differentiated HCC (Grade III and IV) and examined the expression status of 1,706 reported metabolic genes [23]. Aided by hierarchical clustering, we found the aberrant expression of 362 downregulated and 135 upregulated enzymes are enriched in poorly-differentiated HCC in compare to that of well-differentiated HCC and normal liver samples (Figure 1A, and 1E). We further validated these aberrant metabolic enzymes expression in two independent HCC datasets conducted in microarray platforms including GSE50579 (61 HCC and 7 normal samples) [24] and GSE36411 (40 HCC and 40 normal samples) [25] (Figure 1C, 1D, 1F and 1G).

### Functional prediction and prognostic value of aberrant expressed metabolic enzymes in poorly-differentiated HCC

**Figure 2:**
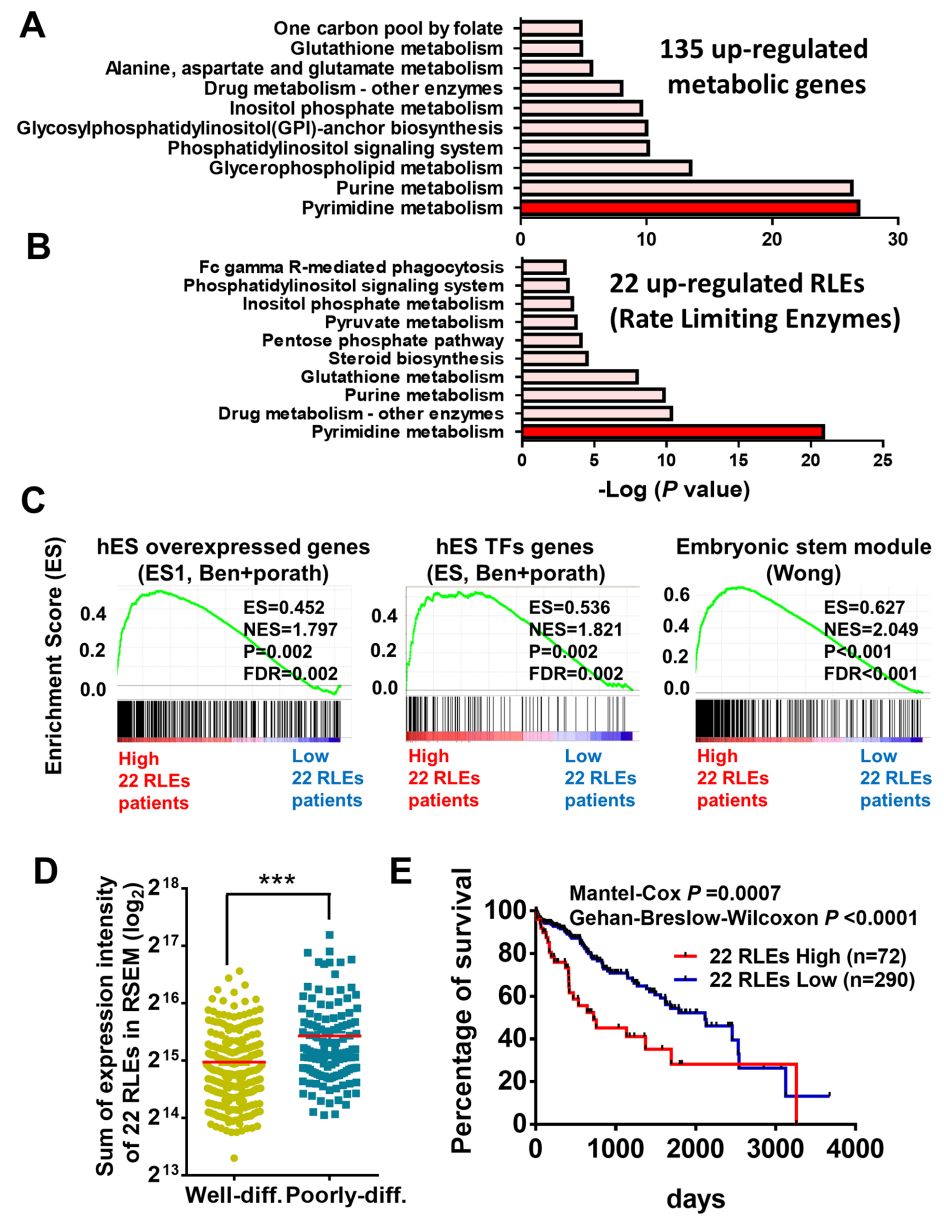
Functional prediction and prognostic value of upregulated metabolic enzymes in poorly-differentiated HCC **(A)** 135 upregulated metabolic genes and **(B)** 22 rate limiting enzymes (RLEs) in the 135 metabolic genes were mainly enriched in pyrimidine metabolism performed in KEGG pathway analysis. **(C)** Upregulated expression of 22 RLEs were associated with stemness gene signatures by GSEA. **(D)** Sum expression intensity of 22 upregulated RLEs is higher in poorly-differentiated HCC than that of well-differentiated HCC in TCGA-LIHC. **(E)** Higher expression of 22 upregulated RLEs is associated with poor survival of HCC patients in TCGA-LIHC. p value was calculated based on Mantel-Cox test and Gehan-Breslow-Wilcoxon test. ***p<0.0001 performed by two-tailed Student's t-test.

To determine the main functions of these aberrantly expressed metabolic genes, we performed pathway analysis matched to the metabolic pathways in KEGG (Kyoto Encyclopedia of Genes and Genomes) database. We found that the upregulated 135 metabolic genes particularly enriched in pyrimidine metabolism and purine metabolism whereas 362 downregulated metabolic genes were enriched in valine, leucine and isoleucine degradation and fatty acid metabolism (Figure 2A and Supplementary Figure 1A). To determine the critical enzymes participated in these metabolic pathways, we further examined the driving rate-limiting enzymes (RLEs) [26] including 22 upregulated RLEs and 77 downregulated RLEs participated in these altered metabolic pathways (Table 1). Consistently, we found that 22 upregulated RLEs (45.4%) were mainly enriched in pyrimidine metabolism and 77 downregulated RLEs (22%) were enriched in fatty acid metabolism (Figure 2B and Supplementary Figure 1B).

**Table 1:** List of 99 rate limiting enzymes in metabolic pathways

	Metabolic pathways	Metabolic rate limiting enzymes
***Nucleotides***	**PYRIMIDINE_METABOLISM**	**UCKL1^a^, TK1^a^, TK2^b^, UCK2^a^, RRM2^a^, RRM1^a^,NT5C3^a^, CAD^a^, TYMS^a^, CTPS^b^, DHODH^b^, DTYMK^a^, CTPS2^a^**
	**PURINE_METABOLISM**	**XDH^b^, IMPDH1^a^, IMPDH2^a^, ADK^b^**
***Lipid***	**FATTY_ACID_METABOLISM**	**ADH1A^b^, ADH1B^b^, ADH1C^b^, ADH4^b^, ADH6^b^, ALDH2^b^,ALDH1B1^b^, ALDH9A1^b^, ALDH7A1^b^, EHHADH^b^, HADH^b^,ACADS^b^, ACOX1^b^, ACOX3^b^, ACSL1^b^, CPT2^b^, ACADL^b^**
	**LINOLEIC_ACID_METABOLISM**	**PLA2G5^b^, PLA2G12A^b^, CYP2J2^b^**
	**ARACHIDONIC_ACID_METABOLISM**	**GGT5^b^, PTGS2^b^**
	**GLYCEROLIPID_METABOLISM**	**PPAP2B^b^, LIPC^b^, LIPG^b^**
	**PRIMARY_BILE_ACID_BIOSYNTHESIS**	**HSD17B4^b^, CYP39A1^b^**
	**STEROID_BIOSYNTHESIS**	**LIPA^b^, SQLE^a^, SOAT2^a^**
	**GLYCEROPHOSPHOLIPID_METABOLISM**	**GPD2^a^, BCHE^b^**
	**STEROID_HORMONE_BIOSYNTHESIS**	**HSD17B6^b^**
***Cofactors and vitamins***	**FOLATE_BIOSYNTHESIS**	**PTS^b^**
	**VITAMINE_B6_METABOLISM**	**PNPO^b^**
	**PORPHYRIN_AND_CHLOROPHYLL_METABOLISM**	**ALAD^b^**
	**RETINOL_METABOLISM**	**CYP3A4^b^, CYP3A43^b^, CYP1A2^b^, CYP2C8^b^, CYP2C9^b^, CYP2C18^b^, CYP2B6^b^, CYP2A6^b^, RDH5^b^**
	**PANTOTHENATE_AND_COA_BIOSYNTHESIS**	**PANK1^b^, PANK4^b^**
***Xenobiotics***	**CAFFEINE_METABOLISM**	**NAT2^b^, NAT1^b^**
***Carbohydrate***	**PROPANOATE_METABOLISM**	**ACACB^b^, ACACA^a^, SUCLA2^b^**
	**PENTOSE_PHOSPHATE_PATHWAY**	**TKT^a^, G6PD^a^**
	**GLYCOLYSIS_GLUCONEOGENESIS**	**DLD^b^, PCK2^b^, PKM2^a^, FBP1^b^**
	**STARCH_AND_SUCROSE_METABOLSIM**	**GYS2^b^, PYGL^b^**
	**AMINO_SUGARS_AND_NUCLEOTIDE_SUGARS_METABOLISM**	**GNE^b^**
	**INOSITOL_PHOSPHATE_METABOLISM**	**PIP5K1A^a^, PIP4K2B^a^**
***Glycans***	**OTHER_GLYCAN_DEGRADATION**	**MAN2B2^b^**
***Amino acids***	**VALINE_LEUCINE_AND_ISOLEUCINE_DEGRADATION**	**HMGCS2^b^, HSD17B10^b^, BCKDHA^b^, BCKDHB^b^**
	**TRYPTOPHAN_METABOLISM**	**OGDHL^b^, IDO2^b^, TDO2^b^, KMO^b^**
	**TYROSINE_METABOLISM**	**TAT^b^**
	**ARGININE_AND_PROLINE_METABOLISM**	**GATM^b^, SAT1^b^**
	**GLYCINE_SERINE_AND_THREONINE_METABOLISM**	**CBS^b^, ALAS1^b^**
	**PHENYLALANINE_METABOLISM**	**PAH^b^**
	**GLUTATHIONE_METABOLISM**	**GSTZ1^b^, MGST1^b^, MGST2^b^, SRM^a^**

a: up-regulated RLEs; b: down-regulated RLEs.

To determine the biological functions of these aberrant metabolic enzymes in poorly-differentiated HCC, we re-classified HCC patients based on the features of histological differentiation and the expression status of 22 upregulated RLEs and 77 downregulated RLEs for gene set enrichment analysis (GSEA) and matched with the annotated functional gene sets collected in the molecular signatures database. No matter the GSEA comparisons were performed in between genes in groups of poorly-differentiated versus well-differentiated HCC ( Supplementary Figure 2A), highly expressed and lowly expressed 22 upregulated RLEs (Figure 2C and Supplementary Figure 2B), and highly expressed and lowly expressed 77 downregulated RLEs (Supplementary Figure 1C and 2C), we found that aberrant gene expression including the metabolic RLEs in poorly-differentiated HCC are strongly correlated with gene signatures of embryonic stem (ES) module [27], human ES cell overexpressed genes and TFs (transcription factors) [28] and proliferation-enhanced G2M checkpoints [29] (Supplementary Figure 2A-2C). These embryonic stemness gene sets were commonly enriched and shared in cancer cells with stemness features and tumor aggressiveness. In contrast, these aberrant metabolic enzymes in poorly-differentiated HCC do not correlate with gene signatures of apoptosis, EMT, hypoxia and inflammatory response (Supplementary Figure 2A and 2C).

We also found that sum of the expression intensity of 22 upregulated RLEs were significantly upregulated in poorly-differentiated HCC than that in well-differentiated HCC in TCGA-LIHC (Figure 2D). On the other hand, the 77 downregulated RLEs were significantly downregulated in poorly-differentiated HCC than that in well-differentiated HCC in TCGA-LIHC datasets (Supplementary Figure 1D). Moreover, when re-classified HCC patients with high RLE score (the sum of all RLE expression value), 77 downregulated RLEs and 22 upregulated RLEs, we found HCC patients grouping with these aberrant RLE expression were associated with poor survival in TCGA-LIHC (Supplementary Figures 1E and 3A, Figure 2E).

### Concordant expression of 99 RLEs and of stemness markers in TCGA-LIHC and six HCC cell lines are correlated with HCC differentiation

**Figure 3:**
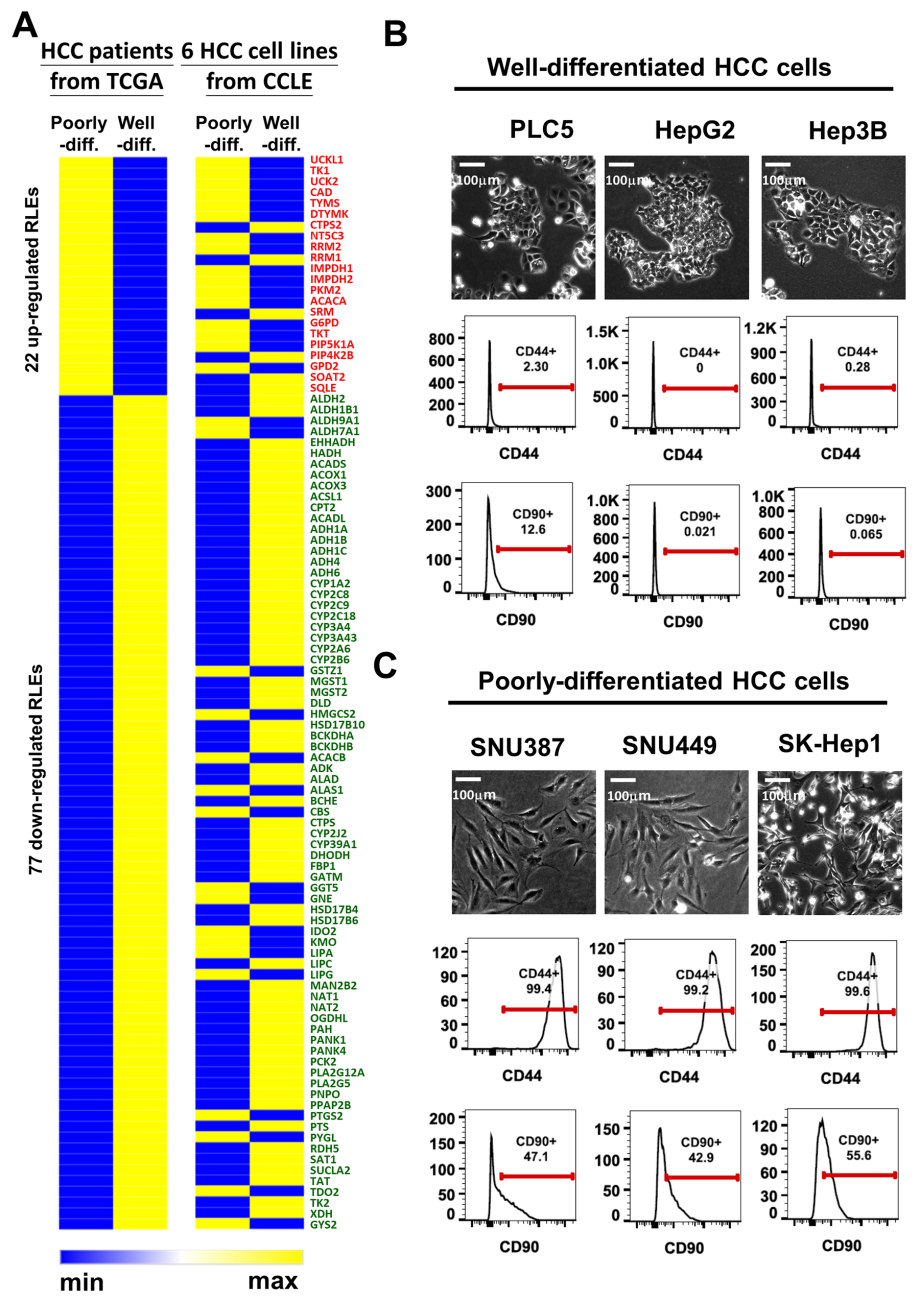
Concordant expression of 99 RLEs and of stemness markers in TCGA-LIHC and six HCC cell lines are correlated with HCC differentiation **(A)** Heatmap clusters of average expression intensity of 99 RLEs in TCGA-LIHC and six HCC cell lines in CCLE. **(B and C)** Cell morphology and expression status of stemness markers CD44 and CD90 in compared with IgG antibody by FACS analysis (B) in well-differentiated HCC cell lines and (C) in poorly-differentiated HCC cell lines. Red lines stand for the gating ranges of CD44+ or CD90+ cells. The numbers under CD44 or CD90 indicates the percentage of cells show positive of CD44 or CD90 in particular HCC cell.

To obtain HCC cell lines with histological differentiation status for functional validation, we downloaded RNA transcriptomic data of six HCC cell lines with known differentiation status, well-differentiated HCC (PLC5, HepG2 and Hep3B) and poorly-differentiated HCC (SNU387, SNU449 and Sk-Hep1) cell lines, from the cancer cell line encyclopedia (CCLE) project. We found high similarity in clusters of the average expression intensity of 99 RLEs expression in compared between TCGA and 6 HCC cell lines by grouping with their differentiation status (Figure 3A). Moreover, we characterized cell morphology and known stem cell markers CD44 [30] and CD90 [31] for confirming their differentiation status. Our results showed that PLC5, HepG2 and Hep3B with epithelial morphology and low CD44/CD90 expression were validated as well-differentiated HCC cells (Figure 3B), whereas SNU387, SNU449 and SK-Hep1 with spindle shape phenotype and high CD44/CD90 expression were confirmed as poorly-differentiated HCC cells (Figure 3C). The associations of 22 upregulated RLEs and 77 downregulated RLEs with high expression of HCC stemness marker CD44 expression were validated in HCC samples of TCGA-LIHC cohort (Supplementary Figure 3B).

### Expression of 10 upregulated RLEs in pyrimidine metabolism pathway are increased in cancer stem cell populations

**Figure 4:**
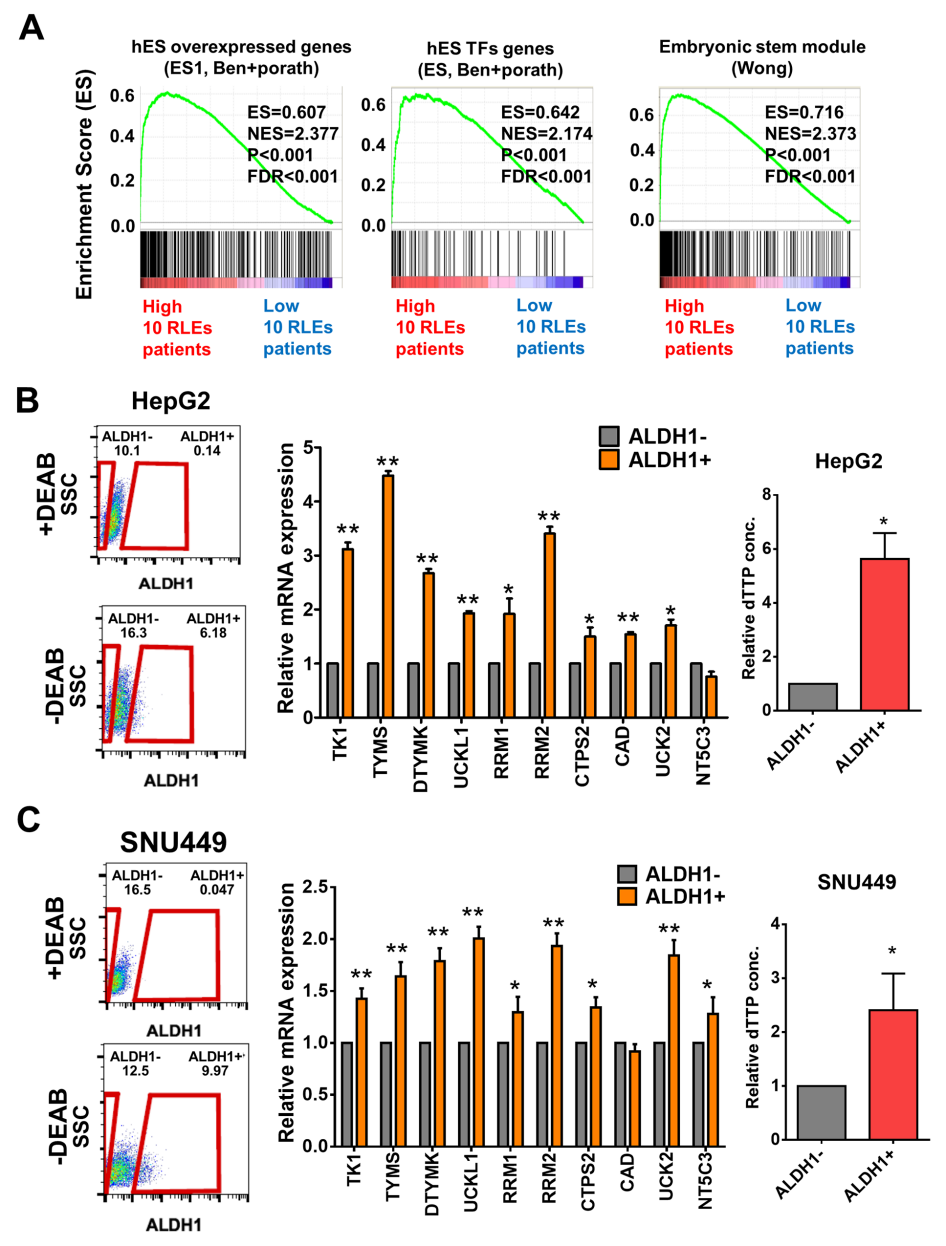
Expression of 10 upregulated RLEs in pyrimidine metabolism in ALDH1 positive stemness sub-populations of HCC cell lines **(A)** Up-regulated expression of 10 RLEs were associated with stemness gene signatures by GSEA. **(B-C)** Higher expression of 10 up-regulated RLEs and relative high dTTP concentration were shown in ALDH1 positive sub-populations than that of ALDH1 negative sub-populations in HepG2 (B) and SNU449 (C) HCC cells. Red boxes indicated the measured areas of ALDH1- or ALDH1+ cell populations. The numbers indicated the percentage of ALDH1- and ALDH1+ population occupied in a given HCC cell in the experiment. *P≤0.05 and **P≤0.001 performed by two-tailed Student's t-test.

Since pyrimidine metabolism was the most enriched pathway in 22 upregulated RLEs in poorly-differentiated HCC with potential prognostic value, we further investigate the expression of 10 RLEs in pyrimidine pathway in stemness population of HCC cells. Firstly, we reclassified HCC patients in TCGA-LIHC with 10 upregulated RLEs in pyrimidine pathway and validated their associations with known stemness signatures by GSEA (Figure 4A). Owing to very low percentage of CD44^+^ and CD90^+^ cell populations in well-differentiated HCC cells (Figure 3B), we isolated cancer stemness sub-populations with the most conservative cancer stemness marker ALDH1 from HepG2 and SNU449 for analysis the expression of 10 RLEs. Our results showed that expression of 10 RLEs of pyrimidine pathway and the relative dTTP concentration were significantly upregulated in ALDH1 positive populations in compared with ALDH1 negative populations of HepG2 (Figure 4B) and SNU449 (Figure 4C) with possibility to participate in cancer stemness properties.

### Three upregulated RLEs in the dTTP biosynthesis of pyrimidine metabolism pathway are essential for stemness and increased cellular dTTP

**Figure 5:**
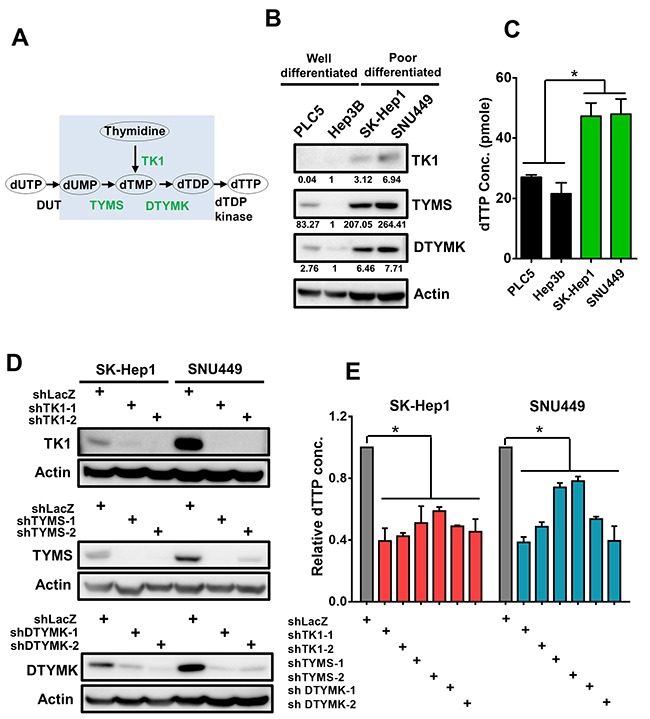
Up-regulated 3 RLEs TK1, TYMS and DTYMK are critical for sustaining cellular dTTP concentration **(A)** TK1, TYMS and DTYMK are rate-limiting enzymes for de novo dTTP synthesis. **(B)** TK1, TYMS and DTYMK are expressed higher in poorly-differentiated than that of well-differentiated HCC cells by Western blotting analysis. The intensity value under a band is normalized to the intensity of actin of the same cell and then divided by Hep3B intensity of the same RLE for comparison. **(C)** Higher concentration of dTTP in poorly-differentiated than that of well-differentiated HCC cells. **(D)** Knockdown efficiency of shRNAs to TK1, TYMS and DTYMK in HCC cells by Western blotting analysis. **(E)** Knockdown TK1, TYMS and DTYMK reduced relative dTTP concentration in poorly-differentiated HCC cells.

Among 10 pyrimidine metabolism enzymes, three RLEs including thymidine kinase (TK1), thymidylate synthetase (TYMS) and deoxythymidylate kinase (DTYMK) were critical for dTTP biosynthesis (Figure 5A). Expression of 3 RLEs in dTTP biosynthesis and the cellular dTTP concentration were higher in poorly-differentiated HCC, Sk-Hep1 and SNU449, than that in well-differentiated HCC, PLC5 and Hep3B by Western blotting and dTTP concentration analysis respectively (Figure 5B and 5C). As shown in our knockdown efficiencies of TK1, TYMS and DTYMK at protein level with shRNAs, we demonstrated that knockdown either one of the 3 RLEs in dTTP biosynthesis pathway decreased the protein expression and the cellular concentration of dTTP in poorly-differentiated HCC cells Sk-Hep1 and SNU449 (Figure 5D and 5E).

### Knockdown 3 RLEs decreased tumor sphere formation, ALDH1^+^ sub-populations and drug resistance to cisplatin treatments

**Figure 6:**
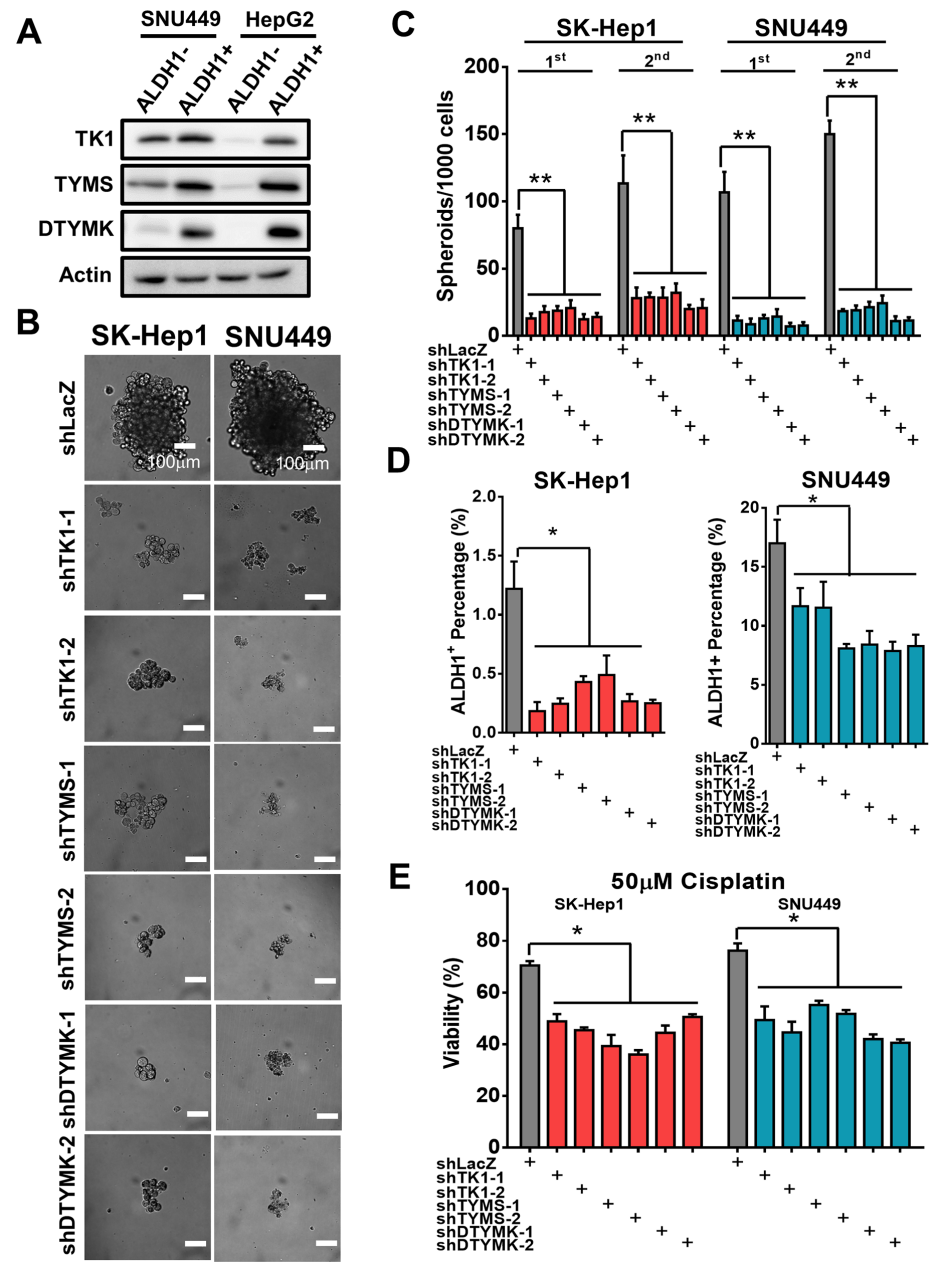
Knockdown TK1, TYMS and DTYMK decreased tumor sphere formation, ALDH1 positive sub-populations and drug resistance **(A)** Higher expression of TK1, TYMS and DTYMK in ALDH1 positive than that of ALDH1 negative HCC cells by Western blotting analysis. **(B)** Knockdown TK1, TYMS and DTYMK reduced spheroids formation ability. **(C)** Knockdown TK1, TYMS and DTYMK reduced tumor spheroid formation in 2 serial passages. **(D)** Knockdown TK1, TYMS and DTYMK reduced expression of ALDH1 stemness subpopulations by ALDHflour analysis. **(E)** Knockdown TK1, TYMS and DTYMK decreased drug resistance to cisplatin treatments.

To examine the participation of 3 RLEs in HCC stemness features, we found that the expression of TK1, TYMS and DTYMK is higher in ALDH1 positive population than that of ALDH1 negative population in HCC cells by Western blotting analysis (Figure 6A). Knockdown of TK1, TYMS and DTYMK reduced capability of forming tumor spheroids as shown in results of microscopy (Figure 6B) and in assays of 2 serial passages (Figure 6C). Moreover, knocking down TK1, TYMS and DTYMK reduce population size of ALDH1 positive cells by ALDHflour analysis (Figure 6D) and resistance to cisplatin treatments in in poorly-differentiated HCC cells (Figure 6E).

### Overexpression of TK1, TYMS and DTYMK increased tumor stemness features and associated with poor HCC patient survival

**Figure 7:**
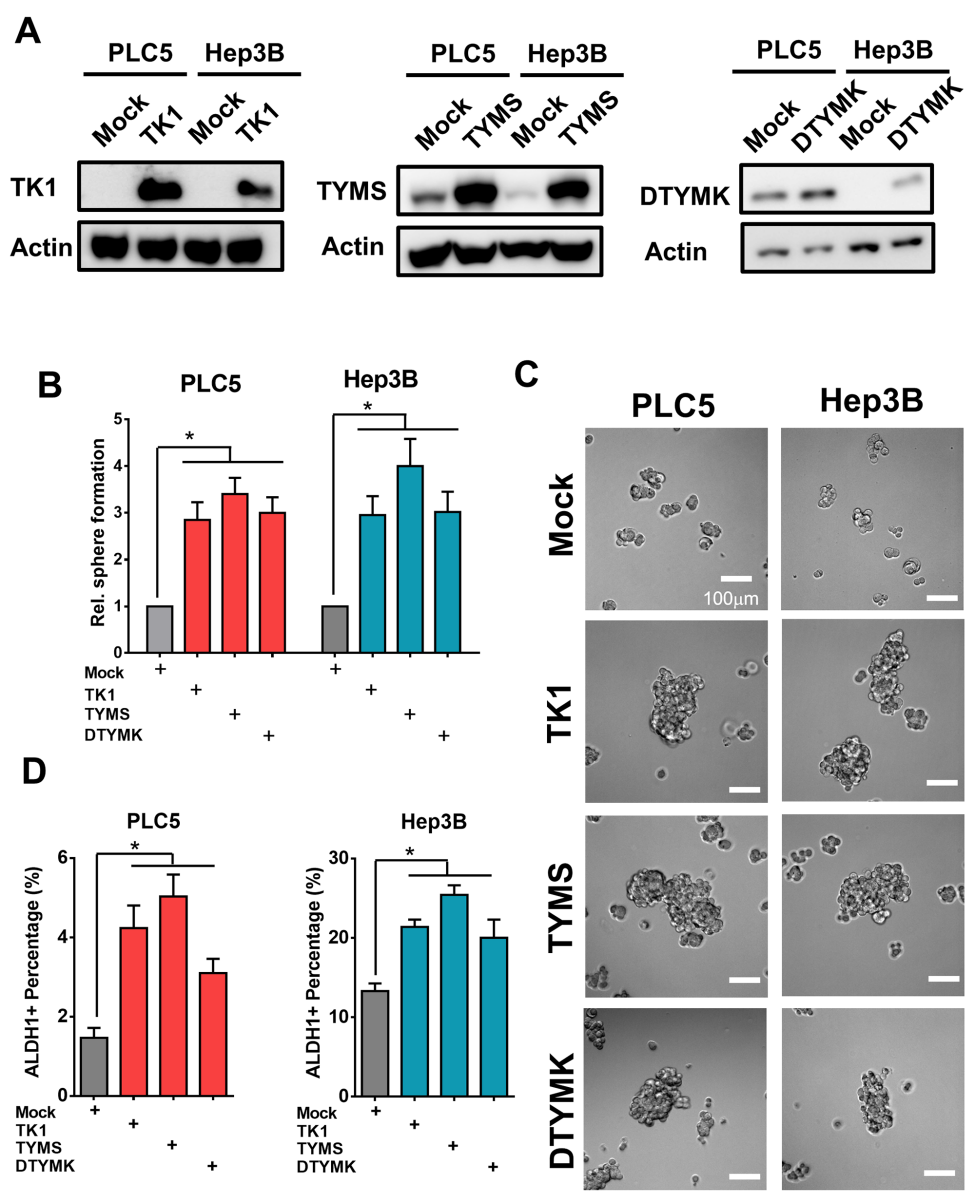
Overexpression of TK1, TYMS and DTYMK increased tumor sphere formation and ALDH1 positive sub-populations **(A)** Overexpression efficiency of TK1, TYMS and DTYMK in HCC cells by Western blotting analysis. **(B and C)** Overexpression of TK1, TYMS and DTYMK increased spheroids formation ability. **(D)** Overexpression of TK1, TYMS and DTYMK increased expression of ALDH1 positive stemness subpopulations.

To further explore the roles of the 3 upregulated-RLEs of dTTP biosynthesis in enhancing stemness features and accompanied with prognostic value, we overexpressed TK1, TYMS and DTYMK in well-differentiated HCC cells PLC5 and Hep3B (Figure 7A). we found that expression of TK1, TYMS and DTYMK enhanced tumor sphere formation capability (Figure 7B and 7C) and increased ALDH1 positive populations in well-differentiated HCC cells (Figure 7D).

**Figure 8:**
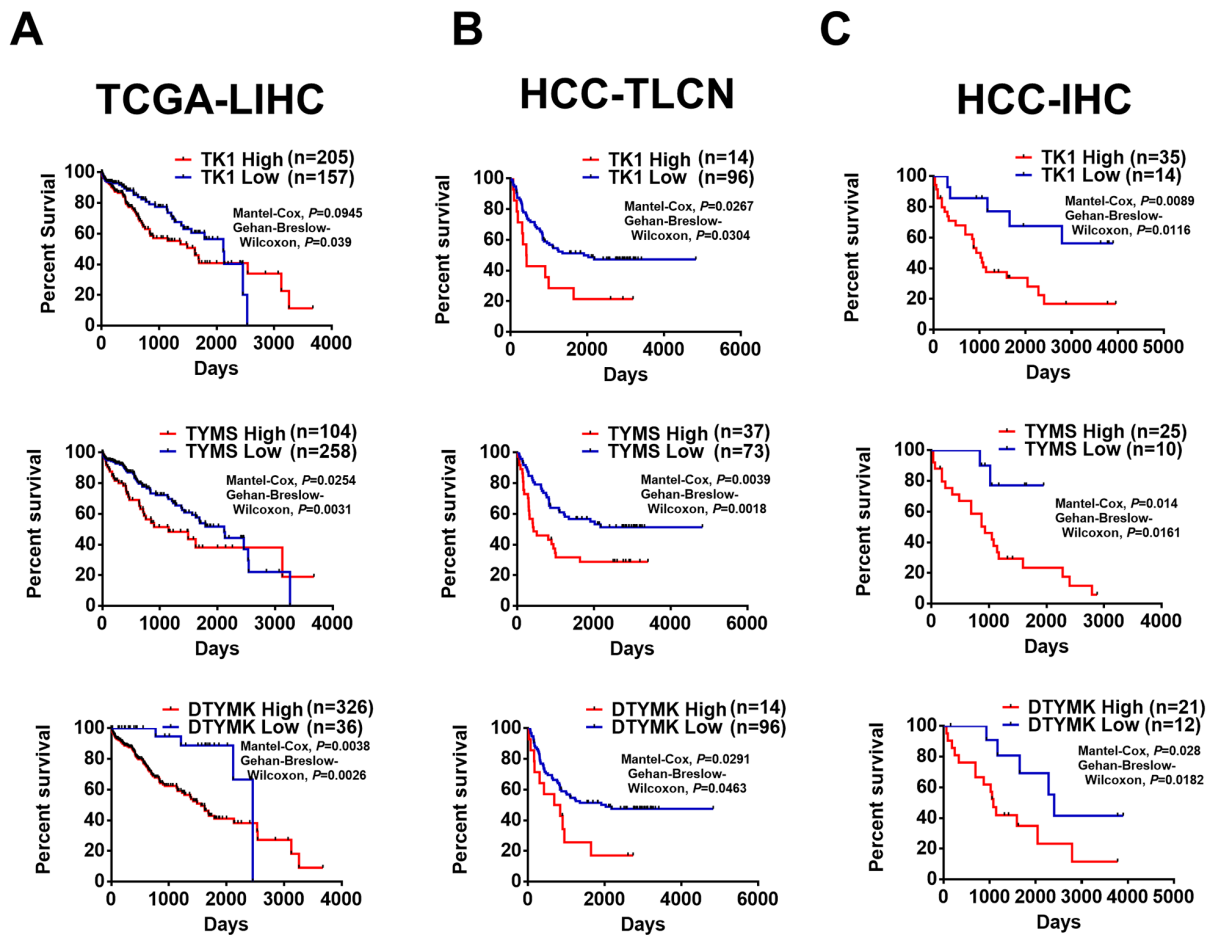
Higher expression of TK1, TYMS and DTYMK is associated with poor patient survival of HCC patients Upregulated TK1, TYMS and DTYMK at RNA level of **(A)** TCGA-LIHC and **(B)** HCC-TLCN (Taiwan liver cancer network), and **(C)** at protein level from HCC tissue arrays by IHC staining (HCC-IHC) are associated with poor HCC patient survival.

Moreover, HCC patients with higher expression of TK1, TYMS and DTYMK at RNA level have poor survival rates in compared to patients with lower expression of 3 RLEs in 362 patients from TCGA-LIHC (Figure 8A). The prognostic value of these 3 upregulated RLEs of dTTP biosynthesis at RNA level expression was further confirmed with 110 HCC patients in Taiwan collected by the Taiwan liver cancer network (TLCN) project [32] (Figure 8B). The prognostic value of 3 upregulated RLEs TK1, TYMS and DTYMK in HCC patients were further confirmed at protein level by performing immunohistochemistry (IHC) on commercial HCC tissue arrays (Figure 8C). Our results of upregulated TK1, TYMS and DTYMK in HCC tumors at RNA and protein levels in multiple independent HCC cohorts further suggested that pyrimidine metabolism especially dTTP biosynthesis pathway is upregulated in poorly-differentiated HCC cells to sustain cancer stemness resulted in poor survival of HCC patients.

## DISCUSSION

To uncover new theranostic targets of HCC, we investigate the roles of tumor metabolism in the lethal aggressive stage, the poorly-differentiated feature in the case of HCC, for exploring the molecular mechanism of tumor malignancy. Through transcriptomic analysis of metabolic enzymes in large HCC cohorts including the RNA-seq data from public TCGA-LIHC and two independent gene expression data based on microarray platforms in GEO, we identified a unique metabolic mRNA signature and candidate cancer genes consisting of 22 upregulated RLEs and 77 downregulated RLEs in poorly-differentiated HCC associated with stemness and poor patient survival. With correlation of expression profiling of metabolic RLEs and differentiation status of TCGA-LIHC and HCC cell lines, we revealed that upregulated RLEs in pyrimidine metabolism especially the 3 RLEs (TK1, TYMS and DTYMK) of dTTP biosynthesis pathway play critical roles in increasing cellular dTTP concentration and sustaining the stemness properties. Upregulated 3 RLEs associated with cancer stemness features might participate in the poor differentiation feature in tumor progression and associate with poor survival of HCC patients.

The major advantage of our integrated genomic approach is to focusing on the aberrant roles of metabolic enzymes in poorly-differentiated HCC and their tumor progression. Consistent with previous tissue metabolomics of high-grade (poorly-differentiated) versus low-grade (well-differentiated) HCC by using NMR spectroscopy, the high-grade HCC showed the increase of lactate, glutamate and alanine and lower levels of lipid, glucose and glycogen in compared with the low-grade HCC [33]. The outcomes of these altered metabolites in high-grade HCC might have resulted from the decrease of RLEs in the amino acid degradation and lipid metabolism and the increase of RLEs in the pyruvate and nucleotide metabolism (Table 1). Our study confirmed the roles of alteration of energy metabolism in HCC histological grading and revealed a novel role of pyrimidine metabolism in supporting stemness and malignant progression of HCC.

The focus of altered metabolism in tumor progression is an emerging field for uncovering new driver genes to target cancer metastasis in recent years. Although previous studies of increased de novo lipogenesis pathway could promote HCC tumorigenesis [34–36], interestingly, recent study to inhibit hepatic lipogenesis by liver-specific knockout of acetyl-CoA carboxylase (ACC) could enhance liver tumorigenesis by increasing antioxidant defense and promoting cell survival [37]. Moreover, high unsaturated lipids were enriched in cancer stem-like cells (CSCs) than that of non-CSC and required for supporting ovarian CSC [38]. Our result of decreased lipid metabolism in association with poor HCC survival is consistent with these recent reports. Nevertheless, another obvious alteration of pyrimidine metabolism pathway in poorly-differentiated HCC for sustaining stemness and association with poor HCC patient survival was never been revealed in previous cancer metabolic studies. Interestingly, approximately half of the 22 upregulated RLEs is involved in pyrimidine pathway, a major contributor to DNA and RNA nucleotide synthesis, to participate in the poorly-differentiated feature, stemness and poor prognosis of HCC.

In the *de novo* pyrimidine pathway, 3 RLEs including TYMS (Thymidylate Synthetase), DTYMK (Deoxythymidylate kinase) and TK1 (Thymidine kinase 1) play critical roles in the dTTP biosynthesis. TYMS, a key rate-limiting enzyme in the folate metabolism, plays essential roles in the development of several malignancies such as prostate cancer and lung cancer [39, 40]. Inhibition of TYMS by treatment with cancer chemotherapy drug 5-FU (5-Fluorouracil) results in accumulation of FdUMP, which might subsequently lead to increased levels of fluoro-deoxyuridine triphosphate (FdUTP) [41]. DNA damage due to FdUTP mis-incorporation results in DNA strand breaks and cancer cell death. DTYMK catalyzes dTTP biosynthesis as synthetically lethal with lkb1 deficiency in mouse and human lung cancer lines [42]. TK1 catalyzes the conversion of thymidine to deoxythymidine monophosphate (dTMP) is function as a proliferation marker in multiple cancer types. In our study, we found that TYMS, TK1 and DTYMK are enriched in ALDH1+ population and knockdown either one of these RLEs decreased ALDH1+ population in poor-differentiated HCC SK-Hep1 and SNU449 cells. Interestingly, upregulation of TYMS and DTYMK was observed in the 5-FU resistant colon cancer cells [43]. Our results of these dTTP biosynthesis RLEs involved in cancer stemness might provide new strategies for 5-FU combination therapeutic modalities for improvement of advanced HCC therapy.

Although cancer metabolism could be influent by intrinsic and extrinsic factors in the tumor microenvironment, understanding metabolic regulation of CSCs might offer new promising approaches for identifying and targeting recalcitrant stem cell populations. CSCs are small but significant populations of cancer cells with self-renewal and tumor-initiating properties. CSCs are known to increase intra-tumoral heterogeneity and drug resistance resulted in disease progression, recurrence, metastasis and adverse patient outcomes [44]. In this study, we revealed pyrimidine metabolic enzymes required for supporting CSC and associated with poor survival of HCC is clinically relevant because many of the enzymes have well-defined active sites that can potentially be targeted by small molecules. Future studies of underlining mechanisms on the metabolic reprogramming of pyrimidine pathway in CSCs and development of drugs to target pyrimidine RLEs should be critical for CSC-targeting therapies with the ultimate goal of overcoming tumor relapse and metastasis.

## EXPERIMENTAL PROCEDURES

### RNA preparation and quantitative reversed transcription PCR (RT-qPCR)

Total cellular RNA was extracted using Trizol reagent (Invitrogen) according to the manufacturer's procedures for RT-PCR. Quantitative RT-PCR was performed by SYBR Green Master Mix (Applied Biosystems) according to the manufacturer's protocols. The specific primers used in RT-qPCR are shown below: hCAD Forward primer (F) 5’ CCCGCAGGAGGACACCTATG, Reversed primer (R) 5’ CGGTGCCCTTCACTTTCTGC; hCTPS2, F: CCGGGAAGCGTGGAGTTCA, R: TGCTGCTGGC AATGATCCCT; hDTYMK, F: GCTGGGAACAAGTG CCGTT, R: ACCAGGTCGGGTTTGGGAAG; hNT5C3, F: CTCTGGGATCCCGCGCTT, R: CTCATGCGCGTCC AAGCAG; hRRM1, F: TTGGATTGTTGCGCCTCTGC, R: CAAGACTGGACTGCGGCTCT; hRRM2, F: GATG AGCCGCTGCTGAGAGA, R: TCTCTCCTCGGGTT TCAGGGA; hTK1, F: TCTCGGGCCGATGTTCTCAG, R: GGTGTTCCGGTCATGTGTGC; hTYMS, F: ACC CTGTCGGTATTCGGCAT, R: AAGTCTCGGGATCC ATTGGCA; hUCK2, F: CGGCAAGTCTTCCGTGTGTG, R: AAGGCATCCGGGTGGTCAAA; hUCKL1, F: ACCA GTCGCGACGAGTTCAT, R: ACACCGGTGATCTG CTTCCC; GAPDH, F: TGTTCGACAGTCAGCCGC, R: GGTGTCTGAGCGATGTGGC;

### Western blotting

Total cellular proteins were extracted by RIPA lysis buffer and then quantified by Bradford method (Sigma). The protein lysates were separated on SDS-PAGE, electro-blotted onto PVDF membranes (Millipore), probed with primary antibody followed by HRP-conjugated secondary antibody, and then detected by enhanced chemiluminescence (ECL).

### *In silico* detection of expression of metabolic genes in cancers

The expression status of metabolic enzymes in liver cancer (LIHC) patients of TCGA was obtained from The Cancer Genome Atlas project (TCGA, https://tcga-data.nci.nih.gov/tcga/). The transcriptome datasets of HCC performed in microarray were downloaded from GEO database. Gene expression was quantified using RSEM (RNA-Seq by Expectation-Maximization) [45] and probe intensity for RNA-seq and microarray datasets, respectively. Quantified RNA expression in TCGA is using RSEM a generative model to estimate RNA expression by EM algorithm and available for download. RLE score was calculated based on the sum of expression value of 22 upregulated RLEs or the sum of expression value of 77 downregulated RLEs in TCGA. The two populations of HCC patients with high and low RLE scores were further classified and performed the gene set enrichment analysis (GSEA) and survival analysis by Cutoff finder [46].

### Spheroid formation assays

Briefly, 1,000 cells were suspended in DMEM/F12 medium containing 20 ng/ml EGF, 20 ng/ml basic FGF and B27 supplements. Cells with limiting dilutions were cultured in 12-well plates for 2 weeks. Spheroids larger than 100 μm were then counted for spheroid-forming index.

### Chemo-resistance assays

10^4^ cells were cultured in the absence or presence of 50μM Cisplatin (Sigma) for two days. The cell viability was further analyzed by PrestoBlue® Cell Viability Reagent (Invitrogen).

### Whole-cell dTTP extraction and quantification

10^6^ cells were extracted with 60% ice-cold methanol, immersed at 100°C dry bath for 3 min and then dried under vacuum according to the method described [47]. The dry residual was further dissolved in 80 μl of nuclease-free water and then detected dTTP levels by following Ferraro et al procedure [48].

### Immunohistochemistry

HCC tissue arrays were deparaffinized and subjected to 10 mM citrate buffer (pH6.0) by microwave treatment for 20 minutes for antigen retrieval. The samples were subsequently immersed in 3% H_2_O_2_ for 30 min to block endogenous peroxidase, and then incubated with primary antibodies of TK1, TYMS and DTYMK diluted in blocking buffer at 4°C overnight. The slides were processed using EnVision+Dual Link System-HRP kit (DAKO) according to the manufacturer's protocol, and counterstained using hematoxylin. Tissue arrays were purchased from SUPER BIO CHIPS (www.tissue-array.com, Seoul, Korea). All IHC results were examined and scored from 1 to 4 based on their expression intensity by two independent pathologists and defined the intensity score above 3 as high level protein expression.

### Flow cytometry analysis for ALDH1 stem cell population

For Aldefluor assay, 5×10^5^ cells were suspended in ALDEFLUOR assay buffer containing ALDH1 substrate according to manufacturer's instructions (Stem Cell Technologies, Durham, NC, USA).

### Gene set enrichment analysis (GSEA)

GSEA was performed on various gene signatures by comparing gene sets from MSigDB database or from published gene signatures [49]. Gene sets with a false discovery rate (FDR) value <0.05 by comparing the enrichment score to enrichment results generated from 1,000 random permutations were considered as statistical significance.

### Statistical and survival analysis

Data was expressed as the mean ± SD. All statistical analyses were conducted using Student's t-test by the SPSS statistical software program (v17.0; SPSS Inc.). Statistical significance was set at *P≤0.05, **P≤0.001 ***p<0.0001 by two-tailed Student's t-test. The chi square test was applied to evaluate the correlation between RLE score and CD44 expression. The high and low populations in RLE score and CD44 expression was selected by first and fourth quantiles respectively. The survival analysis was assessed with cutoff finder at the website http://molpath.charite.de/cutoff/index.jsp [46].

### Antibodies, cell lines and plasmids

Antibodies used in our experiments are TK1, TYMS and DTYMK (Genetex); β-actin (Santa Cruz Biotechnology). Human HCC cell lines PLC5, HepG2, Hep3B, SNU387, SNU449 and SK-Hep1 were maintained in low passage culture as previous described [50]. The full-length TK1, TYMS and DTYMK cDNAs were cloned into pCDNA3.1+ plasmid and deposited into addgene as 100544, 100545 and 100546, respectively.

### Small hairpin RNA and lentiviral infections to cells

The small hairpin RNAs (shRNAs) for TK1, TYMS and DTYMK were obtained from the TRC library: TRCN0000010135 and TRCN0000318729 as *shTK1*; TRCN0000291719 and TRCN0000291720 as *shTYMS*; TRCN0000199082 and TRCN0000199534 as *shDTYMK*; from the National RNAi Core Facility Platform of Academia Sinica. Lentiviral preparation and virus infection were performed as previous described [50]. In brief, pLKO.1 with shRNA, pMD.G and pCMV-ΔR8.91 were introduced into HEK293T cells for lentiviral packaging. The viral supernatants were collected and used to infect HCC cancer cell lines. Control vector expressing shRNA against LacZ (pLKO.1-shLacZ) was used as a negative control.

## SUPPLEMENTARY MATERIALS AND FIGURES


