# Supplementary material for: Pyrimidine metabolic rate limiting enzymes in poorly-differentiated hepatocellular carcinoma are signature genes of cancer stemness and associated with poor prognosis
Source: Oncotarget. 2017 Sep 8;8(44):77734–51. doi: 10.18632/oncotarget.20774 (PMC5652811; doi:10.18632/oncotarget.20774)
Supplement: Supplementary file 1 [file oncotarget-08-77734-s001.pdf]

Pyrimidine metabolic rate limiting enzymes in poorly-differentiated hepatocellular carcinoma are signature genes of cancer stemness and associated with poor prognosis

SUPPLEMENTARY MATERIALS

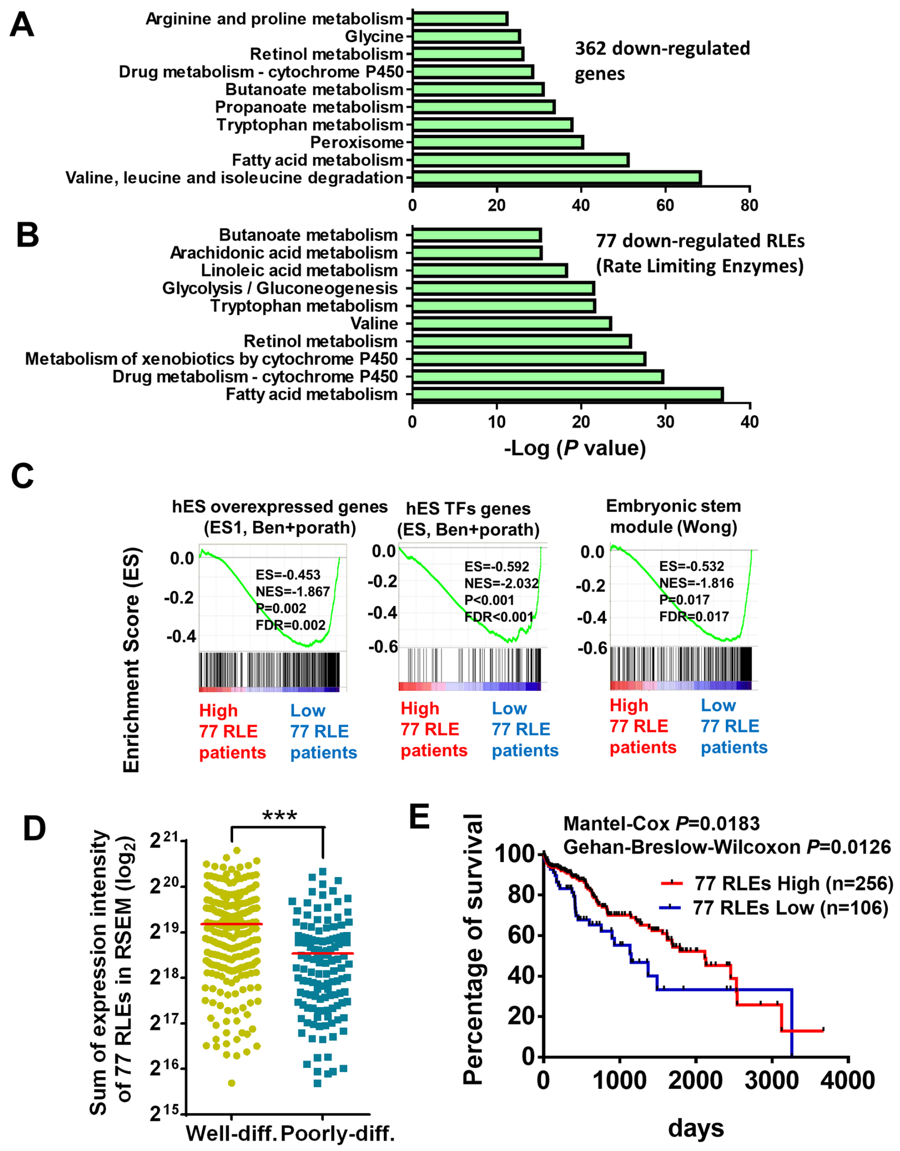

Supplementary Figure 1: Functional prediction and prognostic value of downregulated metabolic enzymes in poorly-differentiated HCC. (A) 362 downregulated metabolic genes and **(B)** 77 rate limiting enzymes (RLEs) in 362 metabolic genes were enriched in various metabolic pathways performed in KEGG pathway analysis. **(C)** Downregulated expression of 77 RLEs were associated with stemness gene signatures by GSEA. **(D)** Sum expression intensity of 77 downregulated RLEs is lower in poorly-differentiated HCC than that of well-differentiated HCC in TCGA-LIHC. **(E)** Lower expression of 77 downregulated RLEs is associated with poor survival of HCC patients in TCGA-LIHC. p value was calculated based on Mantel-Cox test and Gehan-Breslow-Wilcoxon test. ***p<0.0001 performed by two-tailed Student's t-test.

A

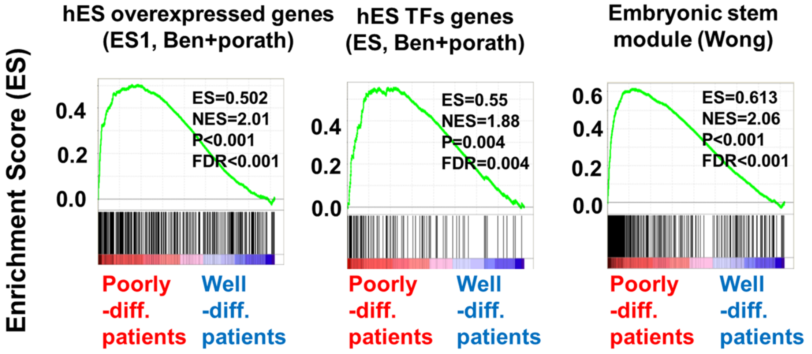

GSEA analysis of genes between well- and poorly-differentiated

GSEA	ES	NES	*P*-val/FDR	Data origin
Embryonic stem module	0.6138	2.0639	<0.001	Wong
hES overexpressed genes (ES1)	0.5024	2.0126	<0.001	Ben-porath
G2M checkpoints (Hallmark)	0.7179	2.1802	0.002	H-MSigDB
hES TFs genes	0.5503	1.8858	0.004	Ben-porath
Hypoxia (Hallmark)	-0.299	-1.216	0.202	H-MSigDB
Apoptosis (Hallmark)	-0.263	-1.11	0.272	H-MSigDB
Epithelial–mesenchymal transition (Hallmark)	-0.219	-0.745	0.696	H-MSigDB
Inflammatory resopse (Hallmark)	-0.183	-0.624	0.897	H-MSigDB

B

GSEA analysis of 22 up-regulated RLEs

GSEA	ES	NES	*P*-val/FDR	Data origin
Embryonic stem module	0.627	2.049	<0.001	Wong
hES TFs genes	0.536	1.821	0.002	Ben-porath
hES overexpressed genes (ES1)	0.452	1.797	0.002	Ben-porath
G2M checkpoints (Hallmark)	0.662	1.949	0.004	H-MSigDB
Inflammatory resopse (Hallmark)	0.303	1.067	0.390	H-MSigDB
Apoptosis (Hallmark)	0.233	1.023	0.391	H-MSigDB
Epithelial–mesenchymal transition (Hallmark)	0.235	0.779	0.675	H-MSigDB
Hypoxia (Hallmark)	-0.229	-0.913	0.572	H-MSigDB

C

GSEA analysis of 77 down-regulated RLEs

GSEA	ES	NES	*P*-val/FDR	Data origin
hES TFs genes	-0.592	-2.032	<0.001	Ben-porath
hES overexpressed genes (ES1)	-0.453	-1.867	0.002	Ben-porath
G2M checkpoints (Hallmark)	-0.666	-2.035	0.006	H-MSigDB
Embryonic stem module	-0.532	-1.816	0.017	Wong
Inflammatory resopse (Hallmark)	-0.291	-1.012	0.452	H-MSigDB
Epithelial–mesenchymal transition (Hallmark)	-0.289	-0.991	0.454	H-MSigDB
Apoptosis (Hallmark)	-0.18	-0.775	0.847	H-MSigDB
Hypoxia (Hallmark)	0.218	0.866	0.616	H-MSigDB

Supplementary Figure 2: GSEA analysis of **(A)** differential expressed genes in poorly- and well-differentiated HCC, **(B)** 22 upregulated RLEs, and **(C)** 77 downregulated RLEs with various gene signatures of stemness, hypoxia, cell proliferation, G2M cell cycle checkpoints, apoptosis, Epithelial-mesenchymal transition (EMT), and inflammatory response.

A

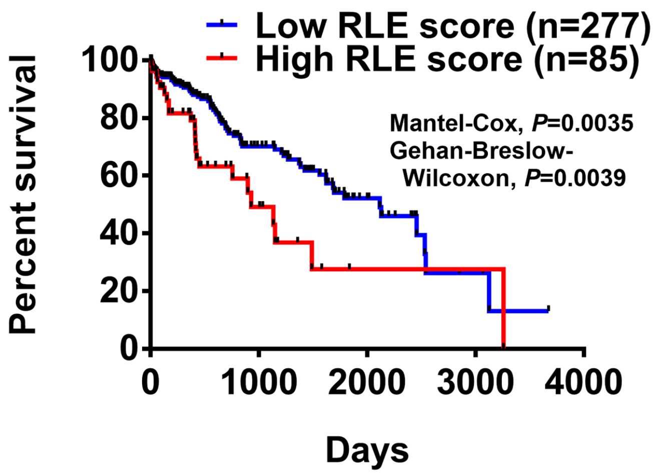

B

	Total (n=179)	22RLEs High	22RLEs Low	*p*-value*
CD44 High	53	35	18	0.0062
CD44 Low	126	55	71	
		77RLEs High	77RLEs Low	
CD44 High	39	12	27	0.0059
CD44 Low	140	78	62	

*estimated by chi square test

Supplementary Figure 3: RLE scoring associated with HCC patient poor survival and high expression of HCC stemness marker CD44. **(A)** High RLE score is associated with poor survival of HCC patients. **(B)** Higher expression of CD44 is associated with high expression of 22 RLEs. In contrast, high CD44 expression is associated with lower expression of 77RLEs in HCC samples of TCGA-LIHC.
